# Mucosal Melanoma: A Rare Entity and Review of the Literature

**DOI:** 10.7759/cureus.9483

**Published:** 2020-07-30

**Authors:** Raman J Sohal, Sandeep Sohal, Ali Wazir, Sam Benjamin

**Affiliations:** 1 Internal Medicine, State University of New York (SUNY) Upstate Medical University, Syracuse, USA; 2 Internal Medicine, The Brooklyn Hospital Center, Brooklyn, USA; 3 Oncology, State University of New York (SUNY) Upstate Medical University, Syracuse, USA

**Keywords:** mucosal melanoma, rare, sinonasal, ipilimumab, nivolumab, asbcopal effect, radiation therapy, chemotherapy, surgical intervention, immunotherapy

## Abstract

Mucosal melanoma is a rare variant of melanoma representing around 1% of total cases of melanoma diagnosed. The usual sites of mucosal involvement are the sino-nasal passages, the oral cavity, and less commonly the upper gastrointestinal (GI) tract. It also has been reported to occur in vulvovaginal and anorectal mucosa.

We present a rare case of mucosal melanoma that presented as recurrent epistaxis, headache, and sinus pressure. CT maxillofacial imaging revealed a large mass right nasal cavity. This was biopsied by ENT and shown to be mucosal melanoma. This was treated with palliative radiation followed by immunotherapy with nivolumab.

Along with details of the case, we also discuss current treatment options with a focus on the role of immunotherapy and its efficacy in cases of head and neck mucosal melanoma. Our review of literature supports use of combination immunotherapy (including both nivolumab and ipilimumab) as it shows greater efficacy than either therapy alone. When combined with radiation therapy (RT) the overall response rate is improved and RT induces an abscopal effect; where benefits of RT are also seen at nonirradiated locations.

In our patient, the use of radiation was essentially palliative as the patient was deemed to not be a surgical candidate. We discuss in our literature review the optimum timing of radiation in relation to definitive surgery or immunotherapy.

## Introduction

Mucosal melanoma is a rare type of melanoma with a very poor prognosis accounting for 1% of all melanomas. This usually occurs in any mucosal surface but most often of the oral, nasal, and sinus tracts as well as the vulvovaginal and anorectal areas [1]. Diagnosis is uniformly made late during the disease course. Typical symptoms are usually due to the localized mass effect. When located in the head and neck region, the symptoms usually include recurrent epistaxis, nasal obstruction, loss of smell, proptosis, limited ocular movement, and headaches. These patients may be misconstrued initially for more common and benign conditions such as migraine or preseptal cellulitis. As noted above by the time the mass is seen on imaging, the tumor had locally advanced and had infiltrated into surrounding structures which produces many of the symptoms seen. Treatment involves wide local excision with negative margins if the disease is localized. However, the anatomical confines of the sinonasal cavities and its lentiginous growth pattern make a complete excision very challenging and morbid [2-4]. Given the advanced presentation in most cases, distant metastasis is common even after resection or other therapy such as immunotherapy and radiation. Therefore, the assessment for metastasis in the initial staging is essential. 

Due to the difficulties with diagnosis, the complicated anatomical location and the infrequency, there have been no large randomized control trials to evaluate for the best treatment approach (although in development) [5]. The five-year survival rate is 25% [6]. Ipilimumab is monoclonal antibody against the CTLA-4 (cytotoxic lymphocyte associated antigen 4). CTLA-4 is a negative regulator of T-cell activation. Thus ipilimumab, being “an inhibitor of an inhibitor” acts to increase T-cell activation and proliferation. The end result is recruitment of additional T-cell with greater activity against immune targeting of the tumor cells. Nivolumab, a humanized monoclonal antibody that blocks PD-1; cell check point inhibitor, also results in increased T-cell function. Due to the novel and differing mechanism of actions, both of these immunotherapies work in concert and independently to attack the tumor cells [4-5].

Recent research has shown that addition of radiation therapy (RT) induces an abscopal and synergistic effect, i.e. in which there is additional anti-tumor action at sites that were not irradiated. This is likely due to be in part from greater tumor antigen presentation to immune cells from the radio-necrosis. Different combinations to exploit this effect are currently in study including: nivolumab + RT, ipilimumab + RT, nivolumab + ipilimumab, nivolumab + ipilimumab + RT [7-8].

## Case presentation

**Figure 1:**
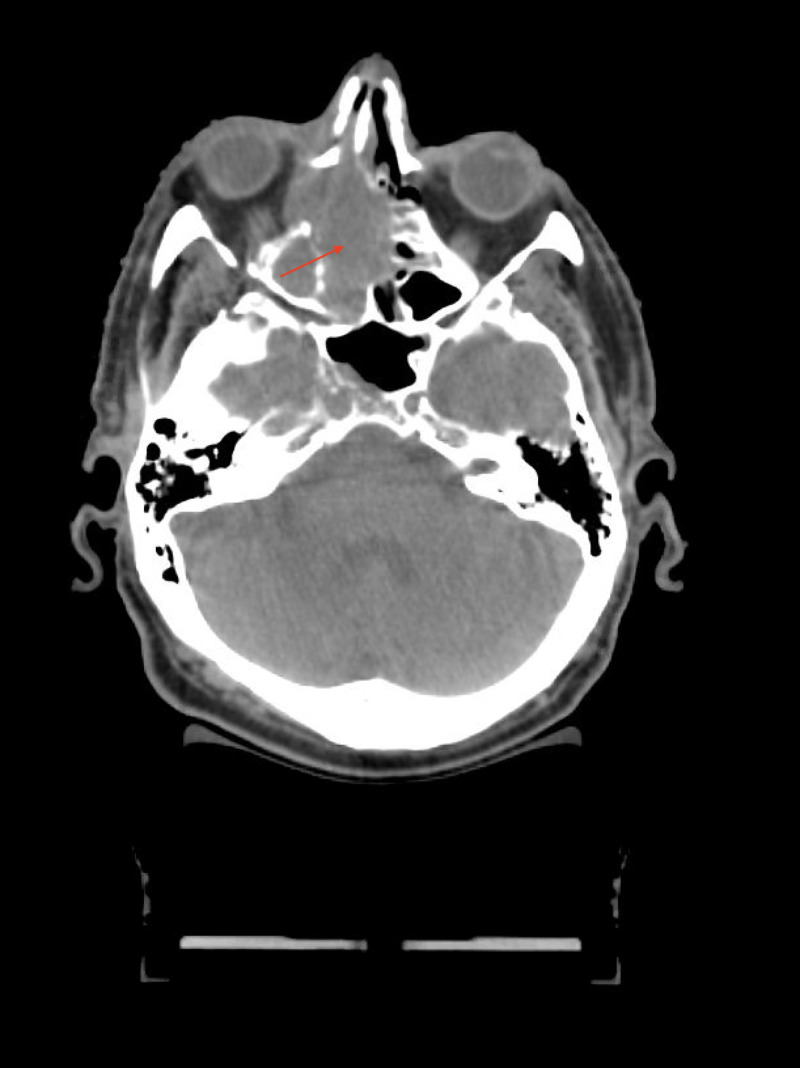
CT head noncontrast showing infiltrative mass in the right nasal cavity eroding through the right lamina papyracea into the right orbit. Red arrow: mass.

**Figure 2:**
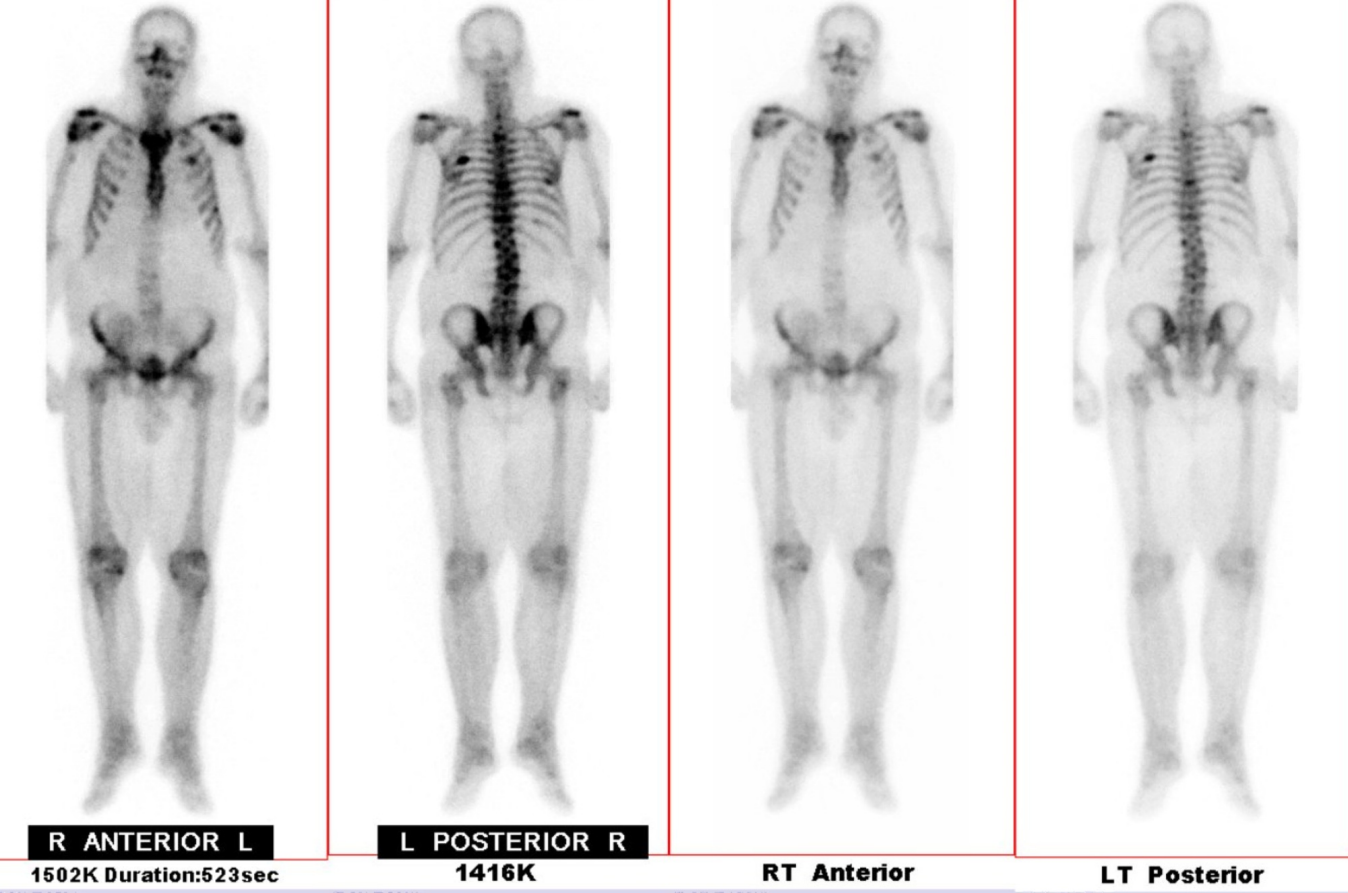
Nuclear bone scan evaluating for metastatic disease. Hyperattenuated regions represent areas of increased radioactive uptake: multiple foci of increased activity which may represent metastatic disease within the lateral left fifth rib as well as the third, eighth, and 11th thoracic vertebral bodies.

**Figure 3:**
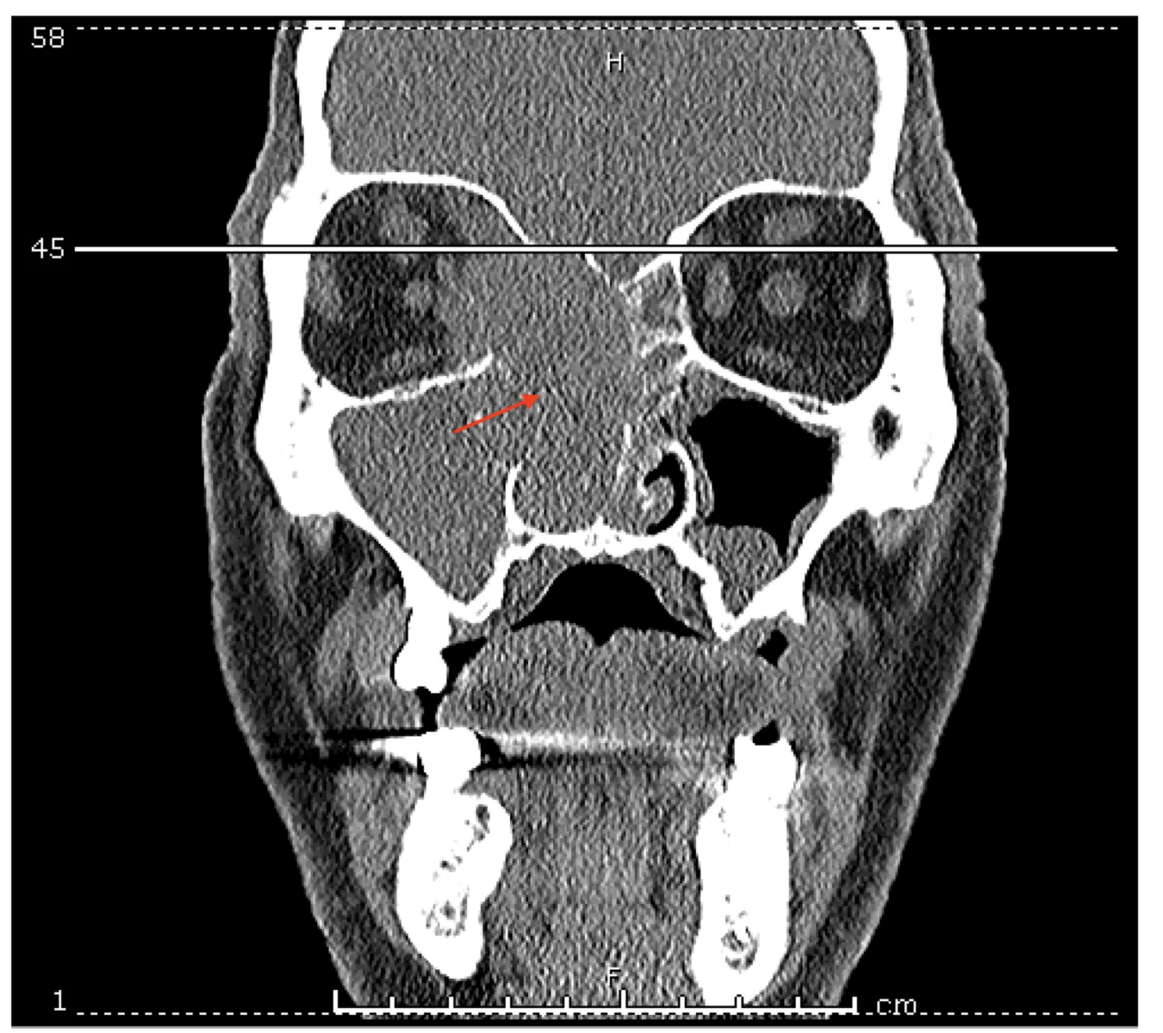
Maxillofacial CT. Red arrow shows 3.5 cm x 1.5 cm mass which has completely obstructed the right nasal cavity and eroded into the nasal septum along with leftward deviation of the septum.

We present a case of a 66-year old male with prior history of prostate cancer (status post prostatectomy) in remission, CKD stage V status post transplanted live donor kidney (in the setting of ESRD secondary to oxalosis) who presented to the hospital with severe right eye pain, proptosis, and pressure for three days. Three months prior he had recurrent headaches and sensation of fullness in the maxillary sinuses. He sought medical attention after his first episode of large volume epistaxis. On inspection of the nares by otoscope a large mass was seen on the right side. CT noncontrast revealed destructive infiltrative mass in the right nasal cavity eroding through the right lamina papyracea into the right orbit (see Figure 1). The mass was biopsied by ENT via a flexible nasopharyngoscopy. Pathology revealed mucosal melanoma. He underwent a positron emission tomography (PET)-CT which showed the primary lesion within the eye/nose with indeterminate nonavid lesions in the left proximal humerus, upper thoracic spine, and liver. Distant metastases could not be excluded. Nuclear medicine bone scan was performed which showed multiple foci of increased activity likely representing metastatic disease within the lateral left fifth rib as well as the third, eighth, and eleventh thoracic vertebral bodies (see Figure 2). The patient was evaluated by a multidisciplinary team consisting of oncology, radiation-oncology, and ENT as patient’s symptoms continued to worsen. A CT maxillofacial scan showed increase in size of the mass since prior imaging. The intraorbital component measured approximately 3.5 cm x 1.5 cm which was increased from prior measurements of 2.7 cm x .8 cm (see Figure 3). The mass now completely obstructed the right nasal cavity and was eroding the nasal septum; along with leftward deviation. Worsening proptosis of right globe was noted. Radiation oncology recommended palliative course of radiation to the orbital/nasal mass to help with controlling his pain and prevent further worsening of his eye site. He was not considered a surgical candidate to the extent of tumor involvement. 

The glomerular filtration rate (GFR) for his transplanted kidney was 15. This was due to chronic rejection and progressive fibrosis despite an adequate immunosuppressive regimen. The standard of care would be using the combination of ipilimumab and nivolumab concurrent with the course of RT. Given the potential for worsening renal function we opted to start with a single agent, nivolumab and palliative radiation. Consideration to start ipilimumab would be made based on how the patient tolerated nivolumab. As planned, he completed five fractions of radiation and was started on nivolumab 480 mg IV monthly along with denosumab for the management of hypercalcemia and pathologic bone fractures. Mutational testing for V600E BRAF was negative. Analysis revealed NRAS mutation with DNA change at position 182 adenine to guanine and amino acid change p.Q61R (Glutamine61Arginine). After his second round of nivolumab therapy the patient developed renal failure with transplant rejection requiring permanent hemodialysis. Four months in therapy the patient did have significant improvement in tumor load regression. Ipilimumab has not yet been started. 

## Discussion

Mucosal melanoma is a rare disease with a poor prognosis. Its hidden presentation on various mucosal surfaces prevents an earlier diagnosis. If diagnosed early, a potentially curative surgical resection can be pursued. Our patient was initially offered craniofacial surgery involving exenteration of the affected eye, removal of portion of the skull with the formation of a skin flap to overlie the resected region. However, this was not recommended by the multidisciplinary treatment team as the ability to produce clear cut-tumor free margins was questionable and the PET-CT images were suggestive of metastatic involvement. Given the patient’s serious comorbid condition including history of renal transplant, it was felt that the patient would not tolerate or recover from this multi-staged surgical procedure. As the patient presented with severe eye pain, proptosis, and blurred vision from mass effect; palliative adjuvant RT was performed to reduce the size of the mass. RT was followed by immunotherapy. The discussion of the evidence comparing mono versus combination therapy is described in a series of trials below and the decision for us to proceed with monotherapy is given [9]. RT has been seen to produce a synergistic effect when combined with immunotherapy as discussed below [10]. 

Evaluation of the data behind monotherapy versus combination therapy in treating mucosal melanoma

A large double-blind randomized control trial performed by Larkin et al. in 2015 assessed the role of combination immunotherapy (nivolumab + ipilimumab) against each of the two monotherapies in managing advanced untreated melanoma. The primary outcome evaluated was progression free survival (PFS). Patients were divided into three separate groups: Group 1: nivolumab + ipilimumab, Group 2: nivolumab, and Group 3: ipilimumab. The PFS was noted to be 11.5 months in the combination group compared to six months for the nivolumab-only group versus 2.9 months for the ipilimumab-only group. The overall response rate (ORR) was 57.7% with the combination group and higher than either therapy individually [9]. 

In the Checkmate 069 trial by Hodi et al. done in 2015 -- the clinical response, PFS, and safety of treatment of advanced melanoma with combination therapy against ipilimumab monotherapy were assessed [11]. Combination therapy resulted in PFS of 8.9 months versus 4.7 months in the ipilimumab-only group. The ORR was 60% in the combination group; a result similar to the above study by Larkin et al. The ORR in the ipilimumab-only group was 11% [9, 11-12].

D’Angelo et al. published a large pooled analysis in 2017 that evaluated the efficacy and safety of combination therapy against nivolumab monotherapy in 889 patients [13]. The median time for PFS in mucosal melanoma was three months with nivolumab alone. When combined with ipilimumab the PFS was increased to 5.9 months [13]. Analysis of grades 3 and 4 adverse effects which include adrenal insufficiency, encephalitis, severe hyperglycemia, Stevens-Johnson syndrome/toxic epidermal necrolysis (SJS/TEN), severe myocarditis or pneumonitis, and colitis was significantly higher in the combination group than monotherapy. The dose of nivolumab used in this trial was 3 mg/kg every two weeks until disease progression or unacceptable toxicity. Alternatively, we used the flat dose of 480 mg every four weeks for one year that could be given when nivolumab was used as a single agent. 

This study also revealed that BRAF mutations were more commonly associated with cutaneous type of melanoma whereas KIT mutations were associated with the mucosal type [13]. Our patient was negative for BRAF and KIT mutations. If the patient had been positive for the V600E BRAF mutation, he could have qualified for BRAF targeted therapy.

The study also showed that PD-L1 expression varied between the different types of melanoma: frequently being less <5% in the mucosal type and >5% in the cutaneous type [13]. PD-L1 has been used a marker for nivolumab activity. This study showed that the ORR was higher in patients with PD-L1 expression >5%; on either mono or combination therapy. However, in patients with mucosal melanoma and PD-L1 expression <5%; the ORR was 33% in patients who received combination therapy. This was suggestive that regardless of the level of PD-L1 expression, nivolumab provided benefit [13]. 

Summary

From the discussion above and a summary of the key studies and their findings, it is clear that combination therapy (nivolumab + ipilimumab) is superior to either type of monotherapy in terms of the ORR and PFS. Also clear from the above, the risks of adverse effects increase with combination therapy. In our patient, we chose to pursue with nivolumab monotherapy. Our rationale was that this would have lesser effect on the patient’s renal status, as ipilimumab is known to be highly nephrotoxic and can induce acute tubular necrosis which may exacerbate his risk of renal transplant failure in an allograft that was already performing poorly from chronic regression (despite being on an extensive immunosuppressive regimen) and progressive fibrosis. The patient was also reluctant to proceed with therapy that may result in transplant failure, although he was aware of the possibility. 

Role of radiation therapy in mucosal melanoma and its effects when combined with immunotherapy

Data show that RT may potentiate the effect of immunotherapy. The radio-necrotic tissue results in increased antigen exposure and thus provides an immune boost. The exact mechanism by which the RT induces immune modulation and synergy with immunotherapy is yet not clear. There are several prospective studies evaluating this synergy published in a review article by Escorcia (2017) [8, 10].

Radiation therapy with each nivolumab monotherapy and ipilimumab monotherapy has been studied individually as well as with combination therapy. In the 2018 retrospective analysis published by Komatsu et al., the degree of tumor regression in patients treated with concurrent nivolumab and RT was assessed [10]. In the study, RT was performed for a median time of five months, with a decrease in tumor size by 42.9% in the maximum diameter. Overall survival at one year was 75% and 50% at two years [10]. Hodi et al. assessed the outcomes of patient on concurrent ipilimumab and RT (varying between 2 and 24 Gy). A substantially longer survival period of 22.4 months versus 8.3 months was noted on concurrent radiation and immunotherapy [11]. This study also revealed that the overall survival was greater if RT was continued during the maintenance period than as neoadjuvant therapy alone (both groups received the same total amount of radiation; however, the group that continued to receive RT through the maintenance period received smaller doses of radiation upfront during the neoadjuvant therapy portion). Study by Postow et al. published in 2020 assessed the role of RT with combined immunotherapy with the main outcome of safety. Overall, the study revealed that combined immunotherapy (niovlumab + ipilimumab) with RT is safe when compared to RT with monotherapy alone [14]. 

In our patient, signs and symptoms related to mass effect including right eye pain, epistaxis, and proptosis started one month prior to diagnosis. The onset to treatment was about 3.5 months from symptom occurrence. We used RT as a neoadjuvant therapy. He received a dose of 2500 cGy in 500 cGy daily fractions to the right maxillary sinus/nasal cavity/right orbit with simultaneous integrated boost gross disease to a dose of 3000 cGy in 600 cGy daily fractions using 6 mV photos (five fractions total). Our patient had received four doses of nivolumab thus far (planned for a total of 12 monthly intravenous injections of 480 mg). He had developed nivolumab-induced vitiligo three months into therapy and had been prescribed topical clobetasol proprionate 0.05% ointment. Initially, the decision to avoid ipilimumab was made because of concern for impairing the patient’s already poor renal status (received a live donor renal transplant from his wife for ESRD secondary to oxalosis). Unfortunately, the patient’s transplanted kidney failed two months into starting nivolumab therapy and he was subsequently started on hemodialysis thrice weekly. He had been receiving regular epoetin injections for anemia (hemoglobin had varied between 6 and 7 g/dL requiring blood transfusions). He had also been started on denosumab to treat hypercalcemia and pathologic bone fractures. Repeat imaging using noncontrast MR face did not show the previously seen mass in the right nasal cavity with extension into right orbit and maxillary sinus. The signal characteristics revealed inflammatory changes and necrotic tumor; however, residual tumor could not be excluded. These are encouraging findings on nivolumab monotherapy with neoadjuvant RT. Overall the patient had a good response to therapy thus far.

## Conclusions

Mucosal melanoma is rare and carries a very poor prognosis. Detection of this cancer is difficult because of tumor growth in hard to access anatomic locations. Frequently, the mass continues to grow becoming noticeable only when it is large enough to press on neighboring structures or invade into surrounding tissue. Surgical intervention with wide local excision and negative margins is thus difficult to attain because of anatomy and delayed diagnosis in which micro-invasion has already occurred. The path of immunotherapy/RT is more commonly taken. Combination immunotherapy (nivolumab and ipilimumab) is superior to monotherapy although the risk of adverse effects is potentially higher. RT has been seen to produce a synergistic effect when used along with immunotherapy in malignant melanoma (regardless of combination versus monotherapy). The mechanism of this immune boost is unclear and currently under investigation. This case report highlights an unmet need of large randomized control trials to define the optimum management of patients with mucosal melanoma. 
